# A solitary primary subcutaneous hydatid cyst in the abdominal wall of a 70-year-old woman: a case report

**DOI:** 10.1186/1752-1947-5-270

**Published:** 2011-07-02

**Authors:** Abdelmalek Ousadden, Hicham Elbouhaddouti, Karim Hassani Ibnmajdoub, Khalid Mazaz, Khalid AitTaleb

**Affiliations:** 1 Service de Chirurgie Viscérale, Hôpital des Spécialités, CHU Hassan II, Route de Sidi Harazem, Fès 30070, Morocco

## Abstract

**Introduction:**

A solitary primary hydatid cyst in the subcutaneous abdominal wall is an exceptional entity, even in countries where the *Echinococcus *infestation is endemic.

**Case presentation:**

We report a case of a 70-year-old Caucasian woman who presented to our hospital with a subcutaneous mass in the para-umbilical area with a non-specific clinical presentation. The diagnosis of subcutaneous hydatid cyst was suspected on the basis of radiological findings. A complete surgical resection of the mass was performed and the patient had an uneventful post-operative recovery. The histopathology confirmed the suspected diagnosis.

**Conclusion:**

Hydatid cyst should be considered in the differential diagnosis of every subcutaneous cystic mass, especially in regions where the disease is endemic. The best treatment is the total excision of the cyst with an intact wall.

## Introduction

Hydatid disease is a parasitic infestation that is caused by *Echinococcus granulosis*, the life cycle of which has been well described [1]. Endemic areas are countries of the temperate zones, where the common intermediate hosts, sheep, goats, and cattle, are raised, such as in North Africa, the Middle East, Central Europe, Australia, and South America [1,2]. The liver is the most frequently involved organ (75%), followed by the lung (15%) [2,3]. The solitary primary subcutaneous localization is extremely rare, and its incidence is unknown [2]. In our patient, the hydatid cyst was located in the abdomen anterior wall without any other involvement, which makes this an interesting case.

## Case presentation

**Figure 1:**
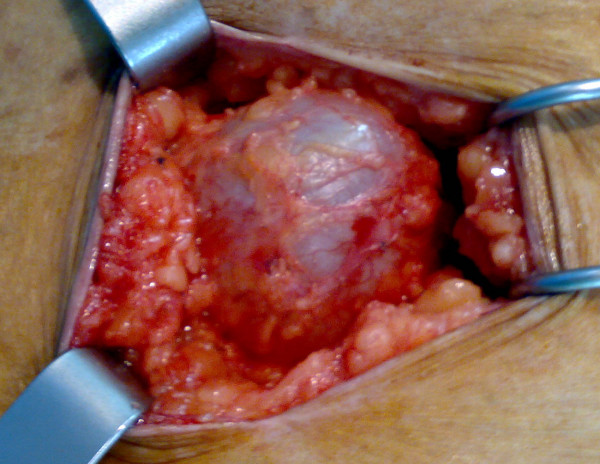
**Peri-operative view of the subcutaneous hydatid cyst**.

**Figure 2:**
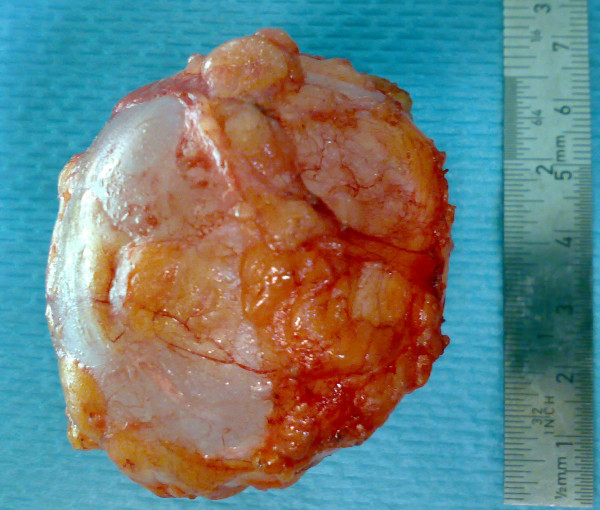
**Image of the totally excised hydatid cyst**.

A 70-year-old Moroccan Caucasian woman presented to our hospital with a subcutaneous cystic mass in the right para-umbilical abdominal wall which had been evolving for six months. Her physical examination revealed an abdominal parietal mass 6 cm in diameter that was palpated 5 cm to the right of the umbilicus. It was cystic, fluctuant, mobile, and painless. The overlying skin was normal. An abdominal ultrasound showed a rounded cystic mass that was limited within the right para-umbilical abdominal wall and measured 60 mm. No other abdominal cystic mass was found. The pre-operative examinations (chest radiograph, complete blood count, urine analysis, and blood biochemistry) revealed no abnormalities. The hydatid serology was negative. Surgical exploration revealed that the mass was attached to the subcutaneous adipose tissue but was not associated with any muscular or cutaneous structure (Figure 1). The macroscopic appearance suggested a hydatid cyst (Figure 2). Perforation was avoided by means of meticulous dissection. The histopathologic examination of the specimen revealed a hydatid cyst. The patient has been followed for two years, and no recurrence of hydatidosis has been detected.

## Discussion

The mechanism of the primary subcutaneous localization is unclear [2,4]. The ingested parasite's ova penetrate the intestinal wall, join the portal system, and reach the liver, where most of them are caught in the hepatic sinusoids [2]. A few ova may pass through the liver (first filter) and reach the lung (second filter) and the systemic circulation, causing hydatid disease in other organs [1,2]. A possible dissemination through lymphatic channels has also been reported. This accounts for cases with solitary cysts in uncommon sites [3-5]. The direct spread from adjacent sites may be another mechanism of infection [6].

In our case, the hydatid cyst was located subcutaneously. The patient had not undergone previous surgery for any hydatid cysts, which were never found in other organs. Therefore, our patient was diagnosed as having a primary subcutaneous hydatid cyst.

In a large series of patients from Greece, the frequency of extra-hepatic and extra-pulmonary hydatidosis was 9% [5]. However, in different series, the frequency of subcutaneous tissue involvement, which is usually associated with involvement of other solid organs, has been reported to be approximately 2% [1,7,8]. Primary isolated hydatid cysts located in the abdominal wall remain extremely rare, however, even in geographic areas in which echinococcal infestation is frequent [3,4].

The clinical course is non-specific and depends on the site of involvement, the size of the cyst, and the pressure caused by the enlarged cyst [1]. Usually, it presents as an inert, painless, non-inflammatory mass without any deterioration of the patient's general condition [4,9]. However, if super-infected or cracked, the cyst can simulate an abscess or a cancer [8,9].

Radiological imaging (ultrasonography, computed tomography, and MRI) is useful in rendering the diagnosis, showing the size, localization, relationship to adjacent organs, and type of the cyst. It can also be used to search for another hydatid location [1,4]. The radiological findings of a thick cyst wall, calcifications, daughter cysts, and a germinative membrane separated from the cyst wall are all specific to hydatid cysts [1-4]. Enhancement of the peri-cystic soft tissues can be considered an MRI feature suggestive of soft-tissue hydatid disease [9]. Serology is a useful tool that confirms the diagnosis, although it is rarely positive for cysts in extra-hepatic and extra-pulmonary locations (25%) [1,4,8]. It is furthermore associated with false-negative and false-positive results [4].

The best treatment option is complete surgical excision of the intact cyst, which avoids leakage of cyst content that can cause anaphylaxis and local recurrence [1,2,8]. If the ideal surgery is impossible, the cyst content (fluid, membrane, and daughter cysts) has to be removed intra-operatively and the cyst pouch has to be irrigated with scolicidal solutions [1,2]. Other options include percutaneous treatment under ultrasound guidance with needle aspiration irrigation of scolicidal solutions, as well as medical treatment with the use of albendazole [2,8].

## Conclusion

Hydatid cyst should be considered in the differential diagnosis of every subcutaneous cystic mass, especially in regions where the disease is endemic. The best treatment is the total excision of the cyst with an intact wall.

## Consent

Written informed consent was obtained from the patient for publication of this case report and any accompanying images. A copy of the written consent is available for review by the Editor-in-Chief of this journal.

## Competing interests

The authors declare that they have no competing interests.

## Authors' contributions

AO, KA, and HE operated on the patient. KHI took the photos. KM participated in follow-up. All authors participated in writing the case report and revising the draft. All authors read and approved the final manuscript.
